# Predicting Caregiver Reactions to Challenging Behaviors in the Context of Dementia

**DOI:** 10.1093/geroni/igab046.3634

**Published:** 2021-12-17

**Authors:** Darby Simon, Benjamin Mast

**Affiliations:** University of Louisville, Louisville, Kentucky, United States

## Abstract

Challenging behaviors exhibited by people living with dementia have been associated with a variety of negative outcomes including greater caregiver burden, nursing home placement, and lower quality of life. Although there has been considerable research on psychological and behavioral changes in dementia, little research has explored family caregiver reactions to these changes and what caregiver characteristics are associated with stronger emotional reactions. This research examined the relationship between established indicators of caregiver mental health (depression, burden, grief, well-being) and caregiver reaction scores on the Revised Memory and Behavior Problems Checklist (RMBPC). The sample consisted of 76 family caregivers for people living with dementia, aged 25 to 93, who participated in a study on caregiver burden and grief. Multiple regression was used to predict RMBPC caregiver reaction scores from the Zarit Burden Inventory, Geriatric Depression Scale, Ryff Psychological Well-Being Scale, and Anticipatory Grief Scale while controlling for RMBPC total behavior frequency scores. RMBPC total behavior frequency scores and Zarit Burden Inventory were significant predictors of caregiver reaction scores (F(2,74) = 87.559, p < .001, R2 = .703). More frequent, challenging behaviors were associated with more distressing reactions and higher caregiver burden also predicted more distress on the RMBPC reaction scores. Psychological well-being was associated with lower reactions at the bivariate level but was not significant in the full regression model. Future research is needed to better understand these relationships and implement this knowledge to benefit family caregivers.

